# Filamentous Fungi in Respiratory Infections. What Lies Beyond Aspergillosis and Mucormycosis?

**DOI:** 10.1371/journal.ppat.1005491

**Published:** 2016-04-28

**Authors:** Anuradha Chowdhary, Kshitij Agarwal, Jacques F. Meis

**Affiliations:** 1 Department of Medical Mycology, Vallabhbhai Patel Chest Institute, University of Delhi, Delhi, India; 2 Department of Pulmonary Medicine, Vallabhbhai Patel Chest Institute, University of Delhi, Delhi, India; 3 Department of Medical Microbiology and Infectious Diseases, Canisius-Wilhelmina Hospital, Nijmegen, The Netherlands; 4 Department of Medical Microbiology, Radboudumc, Nijmegen, The Netherlands; McGill University, CANADA

## Introduction

Respiratory tract infections are globally responsible for one-third of infectious disease–associated mortality, accounting for 4.3 million annual deaths. Among these, fungal infections of the respiratory tract are largely unrecognized, and the true burden is elusive [1]. Despite treatment, most invasive fungal infections are associated with high mortality rates of >50% [2]. In general, fungal infections of the respiratory tract are considered synonymous with invasive pulmonary infections caused by *Aspergillus* spp. and in some centers by Mucorales. However, over the last decade, a number of uncommon filamentous fungi, such as *Scedosporium*, *Fusarium*, *Penicillium*, melanized moulds, and basidiomycetes, have emerged as etiological agents of well-characterized respiratory disorders. It is therefore that the term “respiratory mycosis” has now broadened to include not just invasive disease but also lesser-recognized entities such as fungal ball, severe asthma with fungal sensitization (SAFS), fungus-associated chronic cough (FACC), allergic bronchopulmonary mycosis (ABPM), and allergic fungal rhinosinusitis (AFRS) [3–6]. Notably, both FACC and SAFS have recently been recognized as distinct clinical entities [7,8]. The former manifests as chronic intractable cough in response to pharyngeal colonization by filamentous basidiomycetes, which has been associated with allergic sensitization [7]. The latter, on the other hand, is a reference to poorly controlled asthma, despite optimal management, with evidence of fungal sensitization (short of being labelled ABPM) [6]. *Aspergillus* spp. are considered to be the major culprit of SAFS, although a range of other fungi, such as *Alternaria* and *Cladosporium* spp., are also involved [8]. Both conditions respond favourably to oral antifungal agents, thereby proving a definite role of fungi [9,10]. Moreover, new pathophysiological associations hitherto unknown, such as fungal sensitization and ABPM in patients with chronic obstructive pulmonary disease (COPD), are unfolding [11,12]. Further, emergence of resistance in filamentous fungi to azole antifungal drugs used as mainstay of therapy is another challenging scenario witnessed in the last two decades. This emerging problem is primarily attributed to the widespread usage of azole fungicides in the environment for agricultural and material preservation practices [13]. Here, we aim to provide an overview of the ever-expanding spectrum of human respiratory mycoses and the fungi involved, excluding *Aspergillus* and Mucorales.

## Filamentous Ascomycetes in Respiratory Tract Infections

Several soil-inhabiting genera of the ascomycete order Onygenales, such as *Histoplasma*, *Coccidioides*, *Blastomyces*, and *Penicillium* (*Talaromyces*) *marnefeii*, are thermally dimorphic pathogens, primarily inflicting lungs, while other ascomycetes are at best considered as opportunistic pathogens on a background of underlying local and/or systemic risk factors. The latter group includes most fungi encountered frequently in clinical practice. With the exception of aspergilli, which are the commonest respiratory fungal pathogens, other filamentous fungi causing respiratory diseases include Mucorales, black fungi, and species of *Fusarium*, *Scedosporium*, and *Penicillium*. *Fusarium* spp., conventionally regarded as agents of onychomycosis, are now well known to cause fatal respiratory mycosis. Pulmonary infections are most commonly seen with the *Fusarium solani* species complex, which mimics aspergillosis and is associated with a worse outcome given the resistance to common antifungal agents [14], while *F*. *vasinfectum* is associated with ABPM and hypersensitivity pneumonitis [15,16]. Similarly, species of *Scedosporium apiospermum* complex and *Lomentospora prolificans* (previously *Scedosporium prolificans*) are considered “emerging” human pathogens. Clinically, all organ systems can be infected, although pulmonary infections are the most common [17]. *L*. *prolificans* typically causes infections in immunocompromised patients, which are associated with high mortality. The spectrum of respiratory diseases due to *S*. *apiospermum* ranges from sinusitis, pulmonary fungal ball, ABPM, and pneumonia [17]. *Scedosporium* bears a curious association with cystic fibrosis, in which it is seen as a frequently isolated filamentous mould, second only to *Aspergillus* [17]. *S*. *aurantiacum* (a member of the *S*. *apiospermum* complex) is mainly isolated from patients with cystic fibrosis and other chronic lung diseases. Treatment outcomes with *Scedosporium* spp. are usually poor, with *L*. *prolificans* being resistant to almost all antifungal agents [17].

Also, species of *Penicillium* are emerging over the last few decades as opportunistic lung pathogens. Among these, *Talaromyces marnefeii* is the fourth most common opportunistic pathogen in HIV/AIDS in Southeast Asia. However, other species of *Penicillium* responsible for respiratory infections include *P*. *chrysogenum*, *P*. *citrinum*, *P*. *decumbens*, *P*. *piceum*, *P*. *commune*, and *P*. *purpurogenum*. Recently, *P*. *oxalicum* is recognized as a pulmonary pathogen in patients with chronic respiratory diseases receiving long-term voriconazole therapy [18]. Interestingly, this species exhibits reduced susceptibility to azoles and resulted in breakthrough infections during voriconazole therapy. Also, other *Penicillium* spp. have high minimum inhibitory concentrations of voriconazole [19]. Lately, the epidemiology of invasive pulmonary aspergillosis is also changing, and *Aspergillus terreus* has emerged as the third most important *Aspergillus* species responsible for invasive aspergillosis in some centres in the United States and Europe. Notably, *A*. *terreus* demonstrates poor in vivo and in vitro response to amphotericin B and is associated with higher mortality than more conventional *Aspergillus* spp. [20].

## Filamentous Basidiomycetes (FBM) as Agents of Invasive Respiratory Disease

**Fig 1:**
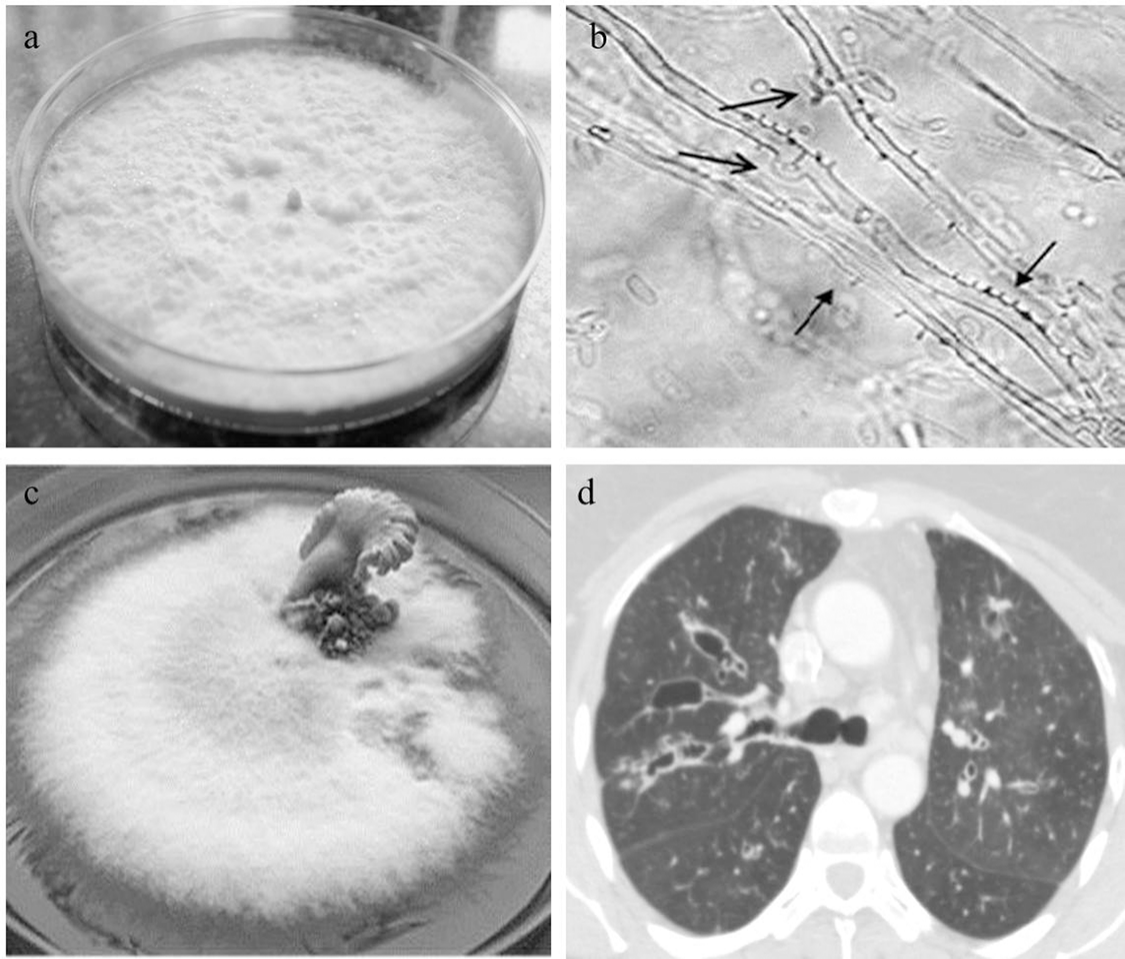
Culture characteristics of *Schizophyllum commune* and computed tomography image of a patient with allergic *S*. *commune* infection. (a) Sabouraud’s dextrose agar petri plate showing white cottony growth of non-sporulating basidiomycete (*Schizophylum commune*) after 10 days of incubation at 28°C. (b) Lactophenol cotton blue mount of slide culture on potato dextrose agar of *S*. *commune* showing clamp connections and spicules after 2 weeks of incubation at 28°C (400x). (c) Plate showing basidiocarp (fruiting body) of *S*. *commune* after 4–5 weeks of incubation at 28°C with periodic exposure to light. (d) High-resolution computed tomography of thorax of patient with allergic bronchopulmonary mycosis due to *S*. *commune* showing central bronchiectasis in both the upper lobes.

The FBM, colloquially called white moulds, have begun to gain importance lately, with systemic studies demonstrating the pathogenic potential of this group of fungi as agents of respiratory and systemic diseases. FBM are usually sterile in culture and appear as cottony white colonies that make routine laboratory identification difficult (Fig 1A). Occasionally, specific characteristics such as spicules, hyphal pegs, clamp connections, arthroconidia, and/or chlamydoconidia may be seen, but they best serve to distinguish FBM fungi from other hyaline ascomycetes (Fig 1B) [3,4,21]. In the past, isolation of these moulds from clinical samples was labelled as contamination; however, lately, several reports incriminating many genera of the phylum Basidiomycota as agents of human disease have been published [4,21]. Notably, FBM such as *Schizophyllum commune*, *Bjerkandera adusta*, *Hormographiella aspergillata*, *Ceriporia lacerata*, *Perennniporia* spp., *Tyromyces fissilis*, and *Irpex lacteus* are described as agents of respiratory diseases [4,5,22–24]. Substantial numbers of white moulds isolated from clinical samples continue to be unidentified, perhaps because of the unavailability of GenBank sequence data for identification [4]. Among the FBM, *S*. *commune* (Fig 1) is probably the best studied, and in a review of 71 infections with *S*. *commune*, 94% were respiratory cases (Fig 1C) [22]. Another FBM, *H*. *aspergillata*, has been reported from Europe and North America as a cause of invasive, predominantly pulmonary disease in patients with haematological malignancies and haematopoietic stem cell recipients and is associated with a high case fatality [4,25]. *Sporotrichum* (*Phanaerochaete*) *pruinosum* is best known for causing chronic lung disease and allergic sensitization in patients with chronic respiratory diseases [4,26]. Lately, a number of newer FBM have been reported as human pathogens. *Perenniporia* spp. and *Ceriporia lacerata* have been incriminated in intracavitary pulmonary fungal ball and fungal pneumonia [23,24]. The list of medically relevant FBM moulds is therefore by no means exhaustive, and with growing awareness among microbiologists and pulmonologists, it will expand in the future.

## Spectrum of Noninvasive Diseases Caused by FBM

FBM are better known as allergens than agents of invasive disease, and their noninvasive respiratory manifestations include colonization, allergen sensitization, and allergic sinopulmonary mycoses. Allergic phenomena involving the lower respiratory tract can manifest as bronchial asthma in atopic individuals, which has been attributed to *S*. *commune* and *Bjerkandera adusta* [27–28] or ABPM due to *S*. *commune* (Fig 1D) [22], while that of the upper respiratory tract manifests as allergic fungal sinusitis [4,22]. Also, sensitization against *S*. *commune* has been reported in patients with COPD, but the clinical implication of this needs to be established [11]. Further, FACC is mainly reported from Japan [5]. The FBM *B*. *adusta* has been shown to colonize the pharynx of susceptible individuals and produce a chronic uneasiness that produces the cough [11]. In FACC patients, FBM have been found in induced sputum cultures [5]. It is pertinent to emphasize here that FBM are slow growers and require prolonged incubation of culture plates for up to 10 days. This is in strong contrast to other filamentous moulds such as Mucorales and *Aspergillus*, which grow within 2 to 3 days of incubation. Thus, the detection of FBM is hampered in microbiology laboratories that discard culture plates of respiratory specimens within 2 days. Furthermore, it has been proposed that a subset of patients with FACC exhibit sensitization to *B*. *adusta*, thereby displaying allergic fungal cough (AFC) that is more severe and difficult to control than the nonsensitized patients with FACC [7]. Low-dose itraconazole has been proposed as therapy for FACC, but the efficacy of this intervention is not unequivocally proven yet [10]. Another unique bronchopulmonary colonization syndrome attributable to *S*. *commune* has been reported exclusively from Japan and involves bronchial impaction of mucus loaded with fungal hyphae [29].

## Black Fungi in the Human Respiratory Tract

Most melanized fungi that cause infections are free-living plant saprobes implanted into human tissue as a result of trauma, but respiratory mycoses occur secondary to inhalation of fungal spores. The moulds implicated in respiratory infections belong to the orders Chaetothyriales (*Exophiala*), Pleosporales (*Alternaria*, *Bipolaris*, *Curvularia*, and *Exserohilum*), Sordiales (*Chaetomium*), and Venturiales (*Verruconis*) of Ascomycota [30]. Among the Pleosporales, *Alternaria alternata* and species of *Bipolaris*, *Curvularia*, and *Exserohilum* are associated with AFRS, bronchial asthma, hypersensitivity pneumonitis, ABPM, and invasive lung disease [15,31–34]. AFRS is a form of polypoid chronic rhinosinusitis caused by type 1 hypersensitivity to fungal antigens. In addition, the disease is characterized by elevated total serum immunoglobulin E, accumulation of thick, eosinophil-laden mucin with noninvasive fungal hyphae within the paranasal sinuses, and nasal polyposis. Although *Aspergillus* species are the most common cause of fungal sinus disease worldwide, allergic fungal rhinosinusitis is more commonly caused by black fungi [35]. Additionally, *A*. *alternata* is known to produce a severe form of asthma through outdoor allergen sensitization [31]. *Chaetomium* spp. are rarely reported as agents of human disease, although *Chaetomium globosum* has been characterized as a cause of fatal pneumonia in patients with haematological malignancies or organ transplants [32,34]. Other respiratory infections attributable to *Chaetomium* spp. include sinusitis and empyema [33]. Also, *Verruconis gallopava* and *Ochroconis* spp. have been isolated from lower respiratory tract secretions and are responsible for invasive and probably allergic lower respiratory tract disease [33,34].

## Therapeutic Challenges and Future Perspectives

The majority of clinical experience with the above-mentioned diverse fungi represents isolated cases or small series of infections. Therefore, evidence-based algorithms for specific treatment are not available and therapy remains a challenge. Corticosteroids, administered systemically or locally, remain the mainstay of treatment for allergic sinopulmonary manifestations. The management of these disorders focuses on three facets. Firstly, as environmental modification is usually impractical, the main focus is to suppress the inflammation with steroids. Alternatively, systemic antifungal agents have been used successfully with the aim of reducing the fungal load albeit best as adjuncts to steroids. Oral itraconazole has proven to be of benefit in disorders such as FACC, SAFS, and ABPM [5,9,15]. Notably, FBM are resistant to echinocandins, and the empirical use of these agents in patients had been associated with breakthrough infections [25]. Also, polyene antifungals like amphotericin B show modest activity against melanized fungi, though some species of *Curvularia* and *Exophiala* could be resistant [30].

To conclude, systematic studies examining the true burden, geographical distribution, and underlying risk factors in patients with respiratory mycoses due to non-*Aspergillus* and mucoralean fungi remains unexplored. Clinical suspicion and broader recognition of FBM-associated diseases among clinicians and microbiologists would improve therapeutic experience and, ultimately, selection of better treatment strategies. It is pertinent to emphasize that thorough identification of non-sporulating moulds in clinical specimens is warranted in order to recognize the clinical entities associated with FBM. Further, prima facie, there appears to be geographical clustering of certain agents and their clinical associations, specifically in Asian countries, which may either be attributable to the expertise and recognition of these agents from clinical samples or other unknown factors that need to be determined. Lastly, to improve the outcome of the diseases associated with these diverse fungi, more studies on pathogenicity, antifungal drug trials, and standardized optimal treatment strategies are warranted.
